# Low-dose tamoxifen treatment in juvenile males has long-term adverse effects on the reproductive system: implications for inducible transgenics

**DOI:** 10.1038/s41598-017-09016-4

**Published:** 2017-08-21

**Authors:** Saloni H. Patel, Laura O’Hara, Nina Atanassova, Sarah E. Smith, Michael K. Curley, Diane Rebourcet, Annalucia L. Darbey, Anne-Louise Gannon, Richard M. Sharpe, Lee B. Smith

**Affiliations:** 1 MRC Centre for Reproductive Health, The Queen’s Medical Research Institute, 47 Little France Crescent, Edinburgh, EH16 4TJ UK; 2 Institute of Experimental Morphology, Pathology and Anthropology with Museum, Bulgarian Academy of Sciences, Sofia, Bulgaria; 3 School of Environmental and Life Sciences, University of Newcastle, Callaghan, NSW 2308 Australia

## Abstract

The tamoxifen-inducible Cre system is a popular transgenic method for controlling the induction of recombination by Cre at a specific time and in a specific cell type. However, tamoxifen is not an inert inducer of recombination, but an established endocrine disruptor with mixed agonist/antagonist activity acting via endogenous estrogen receptors. Such potentially confounding effects should be controlled for, but >40% of publications that have used tamoxifen to generate conditional knockouts have not reported even the minimum appropriate controls. To highlight the importance of this issue, the present study investigated the long-term impacts of different doses of a single systemic tamoxifen injection on the testis and the wider endocrine system. We found that a single dose of tamoxifen less than 10% of the mean dose used for recombination induction, caused adverse effects to the testis and to the reproductive endocrine system that persisted long-term. These data raise significant concerns about the widespread use of tamoxifen induction of recombination, and highlight the importance of including appropriate controls in all pathophysiological studies using this means of induction.

## Introduction

Inducible Cre-lox transgenic systems are widely used to investigate gene function and study cell ontogeny via lineage tracing, as they allow for specific temporal control of genetic manipulation and avoidance of embryonic lethal phenotypes. The most commonly used approach has been to exploit the non-steroidal selective estrogen receptor modulator (SERM) tamoxifen, which has high affinity for estrogen receptors (ER), to create a ligand-dependent Cre recombinase. This Cre recombinase system consists of a fusion protein of Cre recombinase and a mutated form of the human estrogen receptor (CreER^T^)^1, 2^. The CreER^T^ recombinase cannot bind to endogenous estrogens and is only activated when bound to 4-hydroxytamoxifen, which is the active metabolite of tamoxifen^3, 4^; tamoxifen is attractive as an acute and specific inducer compound as it has a half-life of 6 hours *in vivo* ^5^. In the absence of ligand, the CreER^T^ is sequestered in the cytoplasm through heat-shock protein binding. When 4-hydroxytamoxifen enters the cell, it binds to CreER^T^ and displaces the ligand-receptor complex from the heat-shock protein complex, allowing CreER^T^ to translocate to the nucleus and drive recombination of a floxed target gene^3, 4^. Thus, excision of floxed chromosomal DNA is stringently controlled to allow for tight temporal and cell-specific control of genetic modification^6^.

Since the creation of the CreER^T^, newer ligand-dependent recombinases have been developed: CreER^T2^ and mER-Cre-mER, which have an increased efficiency of nuclear import compared to CreERT^3, 7–9^. However, the use of the tamoxifen-inducible Cre-lox system is not without potential drawbacks. Previously published literature has shown that tamoxifen doses commonly used to induce Cre-loxP recombination may continue to label significant numbers of cells for weeks after tamoxifen treatment, possibly confounding the interpretation of time-sensitive studies using tamoxifen dependent models^10–12^. Recombination efficiency of Cre (measured by the number of cells that express a Cre-induced reporter protein) varies with factors such as: the route of tamoxifen administration, the type of transgene, the tissue it is expressed in, mouse age and strain^4^. Recombination efficiency also increases with the dosage^13^ and the number of doses^14^ of tamoxifen administered. However, increasing the dose of tamoxifen can result in increased morbidity^15^, so exploitation of the system becomes an empirically defined compromise between several conflicting factors.

As a SERM, tamoxifen is not an inert compound for the control of Cre-loxP mediated recombination, but an active and well-established endocrine disruptor. SERMs are diverse, non-steroidal compounds with complex tertiary structures that allow them to bind to ERs^16, 17^. Once bound, tamoxifen can elicit either agonistic or antagonistic activity depending on the tissue in question, these differences arising because of differential ER expression, differential expression of co-regulatory proteins or ligand-specific ER conformational change that influence co-regulatory protein recruitment, which together modulate gene transcription^16, 17^. For example, tamoxifen acts as an agonist in the endometrium and the bone, but is a potent antagonist in breast tissue and thus is widely used to treat ER-positive breast cancer^18–20^. Tamoxifen behaves as a partial agonist when acting through estrogen receptor alpha (ESR1) but acts as an antagonist through estrogen receptor beta (ESR2)^21, 22^, thus adding another layer of complexity to the tissue-specific actions of tamoxifen.

The testis is an organ that both responds to estrogen signalling and produces endocrine hormones that impact almost all tissues of the body; it thus has utility as an exemplar organ for the systemic impacts of tamoxifen treatment. Chronic treatment of adult male mice^23^ or rats^24–28^ with tamoxifen results in dose-dependent seminiferous tubule atrophy and therefore decreased fertility. Long term adverse effects on male reproductive function are also well-established to occur after neonatal administration of tamoxifen, including reduced testis and seminal vesicle (SV) size^29, 30^ and a reduction in fertility due to impaired spermatogenesis and decreased seminiferous tubule diameter^31^.

The present study investigates the long term impacts on the testis of a single tamoxifen injection at a range of doses in male mice at pubertal ages, including a dose well below that necessary to induce transgene expression and shows that even a single, low dose, systemic injection of tamoxifen induces persistent adverse changes to both spermatogenic and endocrine functions of the testis that can be detected weeks later. These findings raise concerns relating to confounding effects from the use of tamoxifen-induction of transgenes, and highlight the importance of including appropriate controls in all genetic studies using tamoxifen-mediated transgene induction.

## Results

### Literature review of studies using tamoxifen inducible systems

To assess tamoxifen exposure as a confounding factor in experiments, we completed a PubMed review of the most recent four years of published literature (January 2013 – December 2016) using the search term ‘tamoxifen inducible Cre mouse’. As the technology is now more than 20 years old, this period was chosen both to represent the most current practice, and also because sufficient time and understanding of the caveats of the system has developed to define best practice. This search identified 216 studies, 206 of which are fully accessible from a University of Edinburgh IP address. We assessed these 206 studies (including supplementary data) identifying those that *reported* the inclusion of one or more of six appropriate treatment groups: 1 [Cre positive tam-treated] (41/206); 2 [Cre negative (or floxed alone) tam-treated] (105/206); 3 [Cre positive floxed positive tam-treated] (206/206); 4 [Cre positive vehicle-treated] (13/206); 5 [Cre negative (or floxed alone) vehicle-treated] (27/206); 6 [Cre positive floxed positive vehicle-treated] (64/206).

Whilst the gold standard would be to employ all six controls, we realise that for pragmatic reasons, in particular where the lines used have been well-characterised in previous publications, the absolute minimum controls that should be used when comparing within an experiment are control 1 and/or 2, a non-inducible transgenic mouse exposed to tamoxifen. 121/206 studies reported use of one or both of these a control, leaving 85 primary research studies published in the past four years at risk of undocumented confounders arising from the impact of tamoxifen exposure. Worryingly, 15 of these 85 studies reported Cre positive floxed positive vehicle-treated as the only control, whilst 36 of the 85 did not report or show data from any controls whatsoever.

### Published tamoxifen treatment regimens for transgene induction vary widely

To evaluate the dose range and frequency of tamoxifen administration used in published studies for tamoxifen inducible Cre systems, fifty representative studies from 1998 onwards, covering all ages and dose regimens, were selected from an PubMed query for ‘Tamoxifen inducible Cre mouse’. These 50 studies yielded a total of 85 dose regimens (Supplementary Table 1). This showed that a wide range of tamoxifen doses have been determined as being optimal in individual studies, even when mice were of similar ages; which is presumably dependent on previously determined efficiency of transgene recombination.

To empirically test the long-term impacts of single doses of tamoxifen we chose to inject a single dose of tamoxifen at either postnatal day (d)16 or 21 (early puberty for the mouse), which would permit us to examine both immediate impacts on physiology, but also enable us to track long-term impacts on reproductive development and endocrine function into adulthood. Based on the average dose used in previously published literature (Supplementary Table 1), a single dose of 3 mg tamoxifen was considered to be representative.

### A single dose of 3 mg tamoxifen induces adrenal but not testis transgene expression

**Figure 1:**
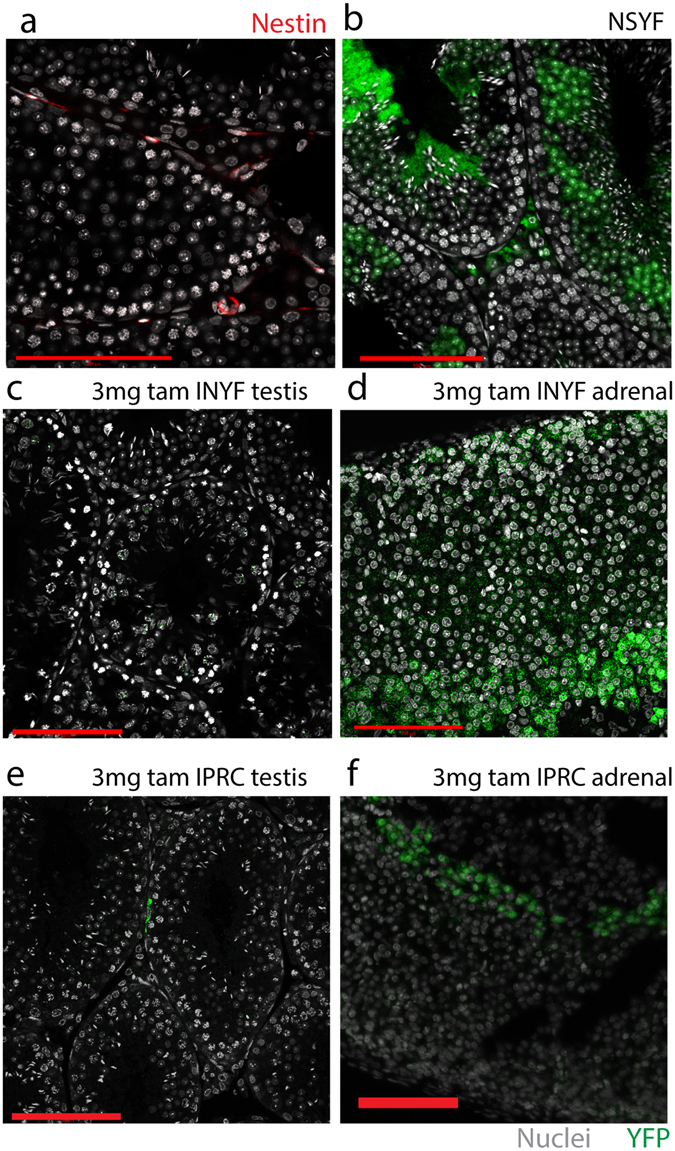
A single dose of 3 mg tamoxifen induces adrenal but not testis transgene expression. (**a**) A population of interstitial cells immunostain for nestin (red) in control testis sections at d35. (**b**) A population of interstitial cells immunostain for YFP (green) in testis sections from NSYF mice injected at d21 and examined at d35. (**c**) No YFP staining is seen in testis sections from d35 INYF mice treated with a single 3 mg dose of tamoxifen at d21. (**d**) YFP staining is seen in adrenal sections from a d35 INYF mouse treated with a single 3 mg dose of tamoxifen at d21 (green). (**e**) Localisation of Confetti fluorescent protein is restricted to small focal areas in d35 IPRC testes treated with a single 3 mg dose of tamoxifen at d21(green). (**f**) Confetti protein is seen in adrenal sections from d35 IPRC mice treated with a single 3 mg dose of tamoxifen at d21 (green). Scale bars are all 100 µm.

We first focussed upon the dose of tamoxifen necessary for the induction of a Nestin-Cre-ER^T2^ transgene in the testis. Nestin is expressed in an interstitial cell population thought to be progenitor Leydig cells^32^ (Fig. 1a). Immunohistochemistry for YFP on testes from a Nestin-Cre YFP reporter line (designated NSYF) revealed YFP staining in interstitial Leydig cells when examined in adulthood (Fig. 1b), which became our positive control for the predicted expression pattern if Nestin-CreER^T2^ was induced. A tamoxifen-inducible Nestin-Cre (Nestin-Cre/ER^T2^) YFP reporter line (designated INYF) was treated with a single 3 mg dose of tamoxifen at d21. This did not induce YFP expression in the testis by d35 (Fig. 1c), however induction of adrenal expression of YFP was found (Fig. 1d), suggesting that 3 mg induces transgene expression in some target organs but not others. To confirm that this was not transgene-specific, we repeated the treatment using a different Cre line known to mark the same cell-types, iPdgfra-Cre/ER^T2^, bred to a different lineage tracing reporter, R26R-Confetti (designated IPRC), with similar results (Fig. 1e,f). On this basis we concluded that this is close to the minimum dose necessary to induce transgene expression in this line.

**Figure 2:**
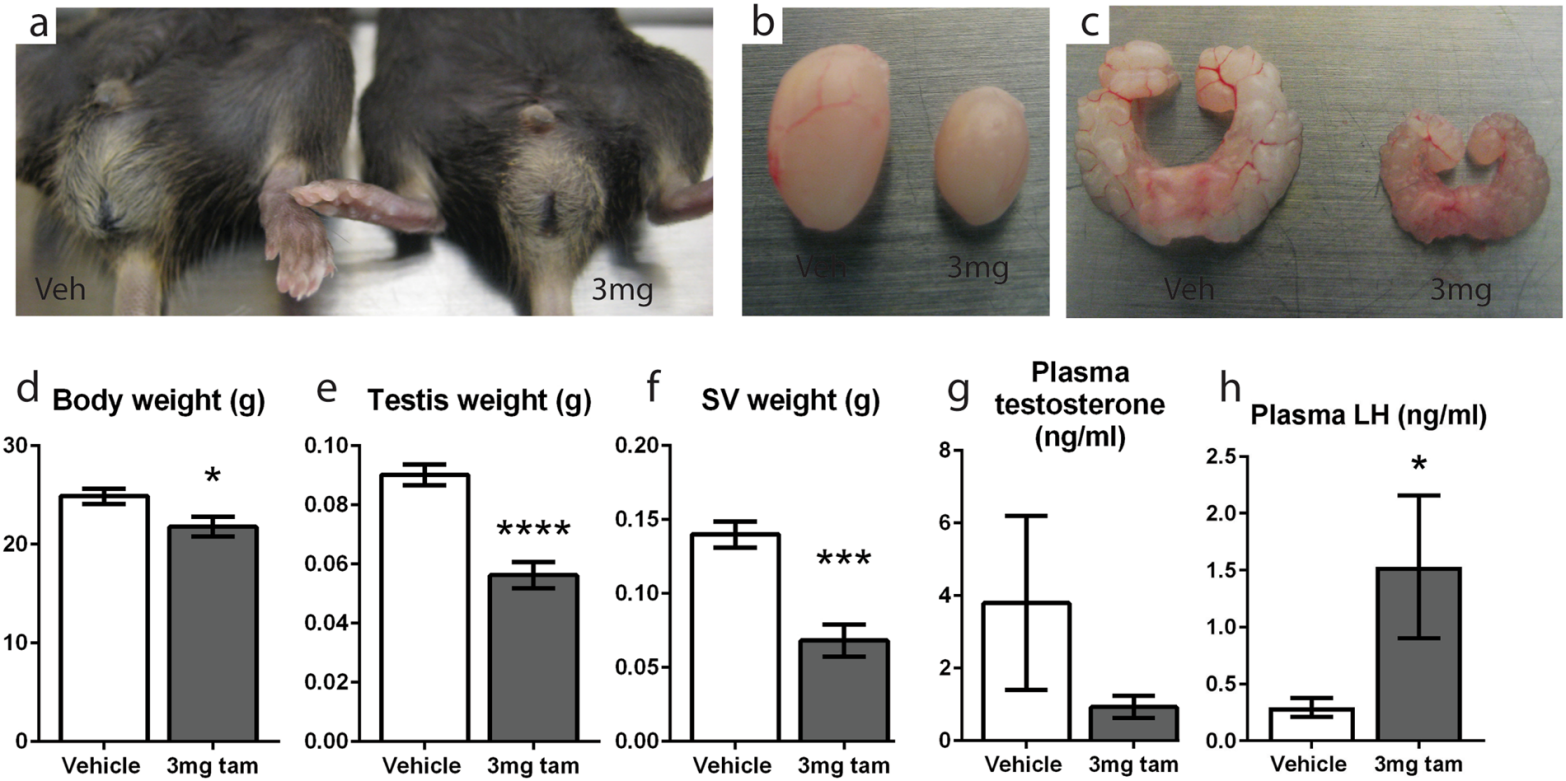
A single dose of 3 mg tamoxifen has long term endocrine effects in male mice. (**a**) INYF mice injected with 3 mg tamoxifen at d21 are cryptorchid at 5 weeks whereas the testes of non-treated INYF controls are not. (**b**) Testes of the d21 INYF tamoxifen-treated mice are visibly smaller than their non-treated littermate controls when examined at 7 weeks. (**c**) Seminal vesicles of the d21 INYF tamoxifen-treated mice are visibly smaller than their non-treated littermate controls when examined at 7 weeks. (**d**) C57bl/6 mice treated with a single systemic 3 mg dose of tamoxifen at d16 have significant reductions in body weight, (**e**) testis weight and (**f**) SV weight when examined at 7 weeks. (**g**) C57bl/6 mice injected with tamoxifen at d16 have no difference in plasma testosterone at 7 weeks compared to the controls. (**h**) Plasma LH is significantly increased in tamoxifen-treated mice. Graph values are group means where n ≥ 7, error bars are SEM.

Despite exposing the mice to a tamoxifen dose that was unable to induce transgene recombination in the testis, all six tamoxifen-treated INYF mice injected at d21 were cryptorchid when dissected at 5 weeks of age (Fig. 2a), whereas the testes of all six INYF vehicle controls were normally descended into the scrotum (p = 0.0022, Fisher’s exact test). However, when mice injected at d21 were examined at 7 weeks of age both all seven tamoxifen-treated and all seven untreated mice had normally descended testes. Despite this, both the testes (Fig. 2b) and seminal vesicles (SV) (Fig. 2c) of the tamoxifen-treated mice were visibly smaller than their non-treated littermate controls at 7 weeks.

### A single dose of 3 mg tamoxifen has long term endocrine effects in male mice

To focus on the effects of tamoxifen and exclude any possible confounding factors arising from transgene induction, our further studies used wild-type C57bl/6 mice. Consistent with data from the INYF line, six out of six C57bl/6 mice injected with 3 mg tamoxifen at d16 were also cryptorchid at 5 weeks of age, compared to descended testes in six out of six vehicle injected mice at this age (p = 0.0022, Fisher’s exact test), whereas testicular descent had occurred by 7 weeks of age in six out of six of both vehicle and tamoxifen injected groups. However, at the latter age, tamoxifen treated mice exhibited significant reductions in body weight (Fig. 2d), testis weight (Fig. 2e), and SV weight (Fig. 2f); SV size is a reliable biomarker of circulating testosterone levels but is also directly sensitive to estrogenic effects^33^. Despite this, circulating testosterone levels were not significantly different in tamoxifen-exposed mice compared to the controls (Fig. 2g), however LH levels were significantly increased, indicating compensated Leydig cell failure (Fig. 2h). Together these data show that a single dose of tamoxifen similar to the concentration usually given to induce a transgene elicits long term adverse effects on testis size and endocrine function five weeks after tamoxifen exposure.

### A single dose of 3 mg tamoxifen has long term effects on spermatogenesis

**Figure 3:**
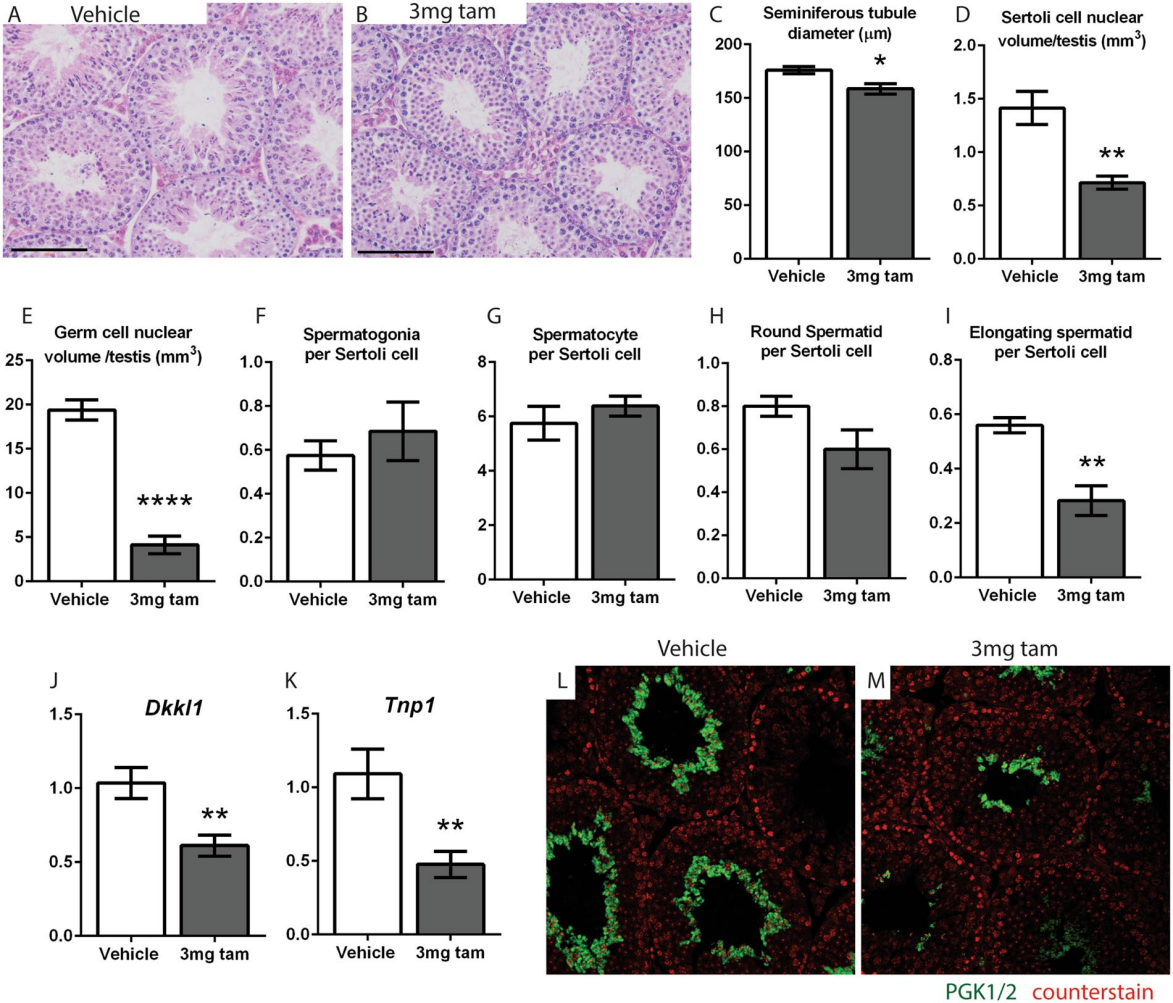
A single dose of 3 mg tamoxifen at d16 has long term effects on spermatogenesis. (**A**,**B**) C57bl/6 mice treated with tamoxifen at d16 have visibly normal testicular histology, with no gross changes to seminiferous tubule organisation at 7 weeks compared to controls. (**C**) The seminiferous tubules of tamoxifen-treated mice have a significantly smaller diameter than controls (n = 3 mice, 50 measurements per testis, error bars are SEM). Stereological cell counting reveals a significant reduction in (**D**) Sertoli cell volume and (**E**) germ cell volume per testis in tamoxifen-treated mice. (**F**–**H**) The number of spermatogonia, spermatocytes and round spermatids per Sertoli cell is unchanged. (**I**) The number of elongate spermatids per Sertoli cell is significantly reduced (cell counting measurements n = 4 mice, error bars are SEM). Testes from tamoxifen-treated mice also show significant reductions in the expression of (**J**) the spermatocyte marker *Dkkl1* and (**K**) the spermatid marker *Tnp1* (qPCRs n = 7, error bars are SEM). (**L**) Testes from tamoxifen-treated mice have a visibly reduced area of immunohistochemical staining for the spermatid marker PGK 1/2.

When examined at 7 weeks of age, mice treated with tamoxifen at d16 had grossly normal testicular histology, with no major changes to seminiferous tubule organisation (Fig. 3A,B). However, the seminiferous tubules of tamoxifen treated mice had a significantly smaller diameter (Fig. 3C) and a significant reduction in both Sertoli cell (Fig. 3D) and total germ cell volume per testis (Fig. 3E). Although the numbers of spermatogonia, spermatocytes and round spermatids per Sertoli cell were unchanged in tamoxifen-exposed animals (Fig. 3F–H), the number of elongate spermatids per Sertoli cell was significantly reduced. (Fig. 3I). Testes from tamoxifen-treated mice also showed significant reductions in testicular expression of the spermatocyte marker *Dkkl1* (Fig. 3J) and the spermatid marker *Tnp1* ^34–36^ (Fig. 3K), as well as a visibly reduced area of immunohistochemical staining for the spermatid marker PGK 1/2 (Fig. 3L). These findings show that a single early pubertal exposure to tamoxifen results in a permanent reduction in Sertoli cell number (and a consequential reduction in total germ cell number). However, in addition to this overall change, tamoxifen exposure resulted specifically in a significant loss of spermatids per Sertoli cell, which will result in a corresponding reduction in number of sperm produced.

### A single dose of 1 mg or 250 µg tamoxifen does not delay testis descent, yet still has persistent effects on the male reproductive system

**Figure 4:**
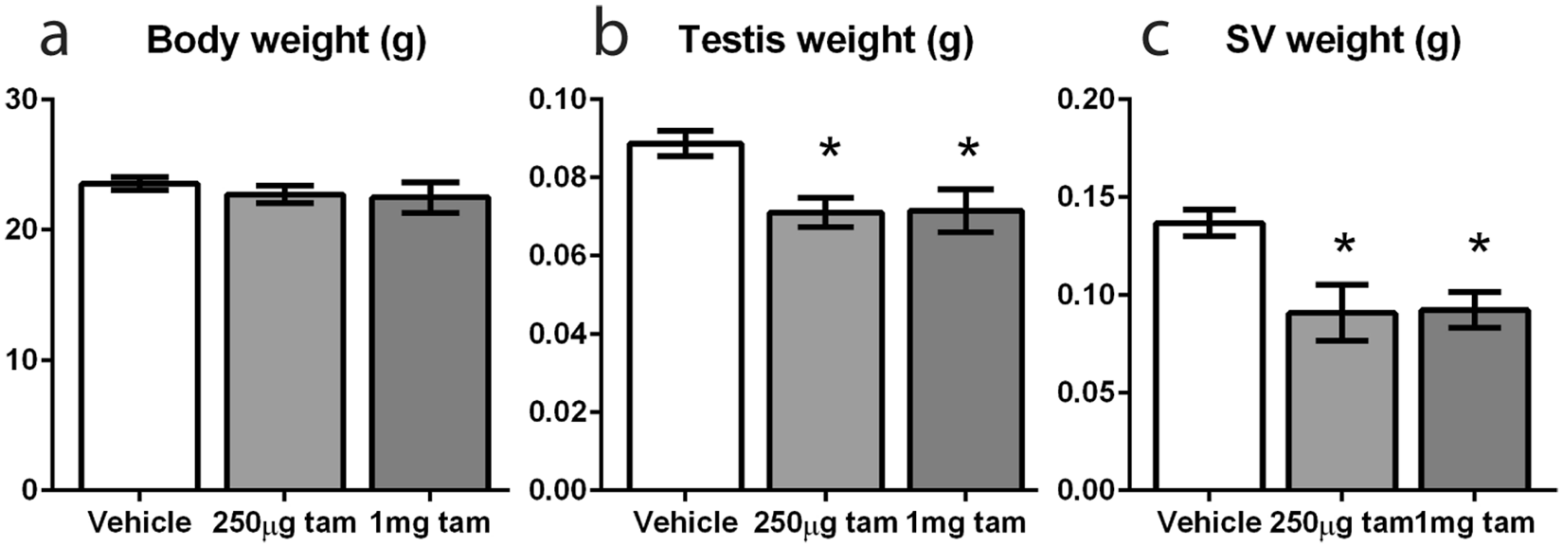
A single dose of 1 mg or 250 µg tamoxifen at d16 does not delay testis descent, yet still reduces testis weight. (**a**) INYF mice treated with either 1 mg or 250 µg tamoxifen at d16 do not have a significant change in body weight compared to controls at 7 weeks. (**b**) Both 1 mg and 250 µg groups have a significant reduction in testis weight compared to controls at 7 weeks. (**c**) Both 1 mg and 250 µg groups have a significant reduction in SV weight compared to controls at 7 weeks. (All measurements n ≥ 6, error bars are SEM).

**Figure 5:**
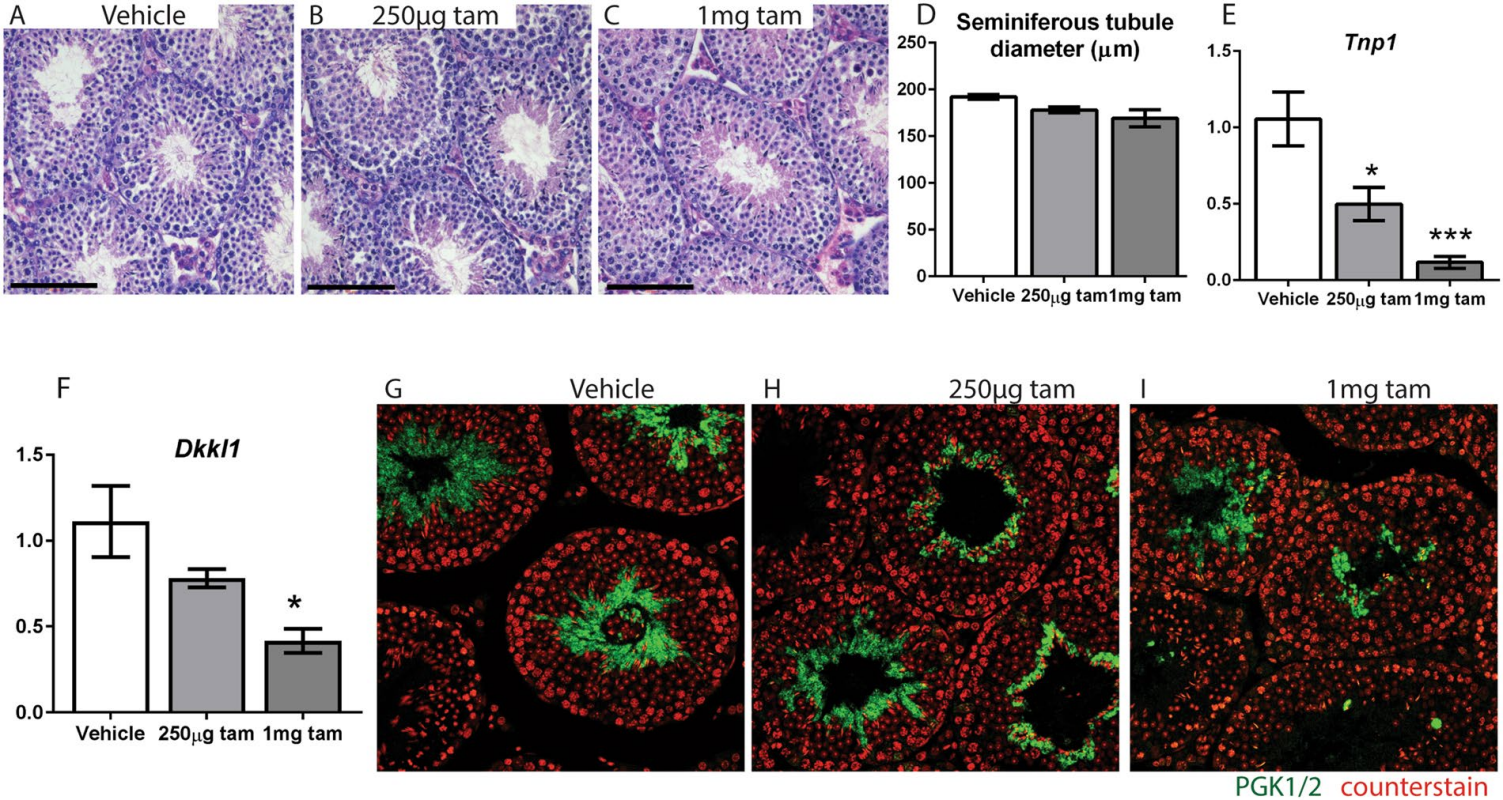
A single dose of 1 mg or 250 µg tamoxifen at d16 has long term effects on spermatogenesis. (**A**–**C**) No visible reduction in spermatogenesis at 7 weeks is observed when testicular histology is examined in mice treated with 1 mg or 250 µg tamoxifen at d16. (**D**) No reduction in seminiferous tubule diameter is observed when testicular histology is examined in mice treated with 1 mg or 250 µg tamoxifen (n = 3 mice, 50 measurements per testis, error bars are SEM). (**E**) Stereological cell counting reveals no significant change in Sertoli cell volume per testis in 1 mg and 250 µg groups compared to control. (**F**) Total germ cell volume per testis is reduced in 1 mg tamoxifen-treated mice but not in 250 µg tamoxifen-treated mice. (**G**–**J**) The number of spermatogonia, spermatocytes, round and elongating spermatids per Sertoli cell is unchanged (cell counting measurements n = 5 mice, error bars are SEM). (**K**) 1 mg tamoxifen-treated testes show a significant reduction in *Dkkl1*, whereas 250 µg tamoxifen-treated testes do not. (**L**) Both 1 mg and 250 µg tamoxifen-treated testes have a significant reduction in *Tnp1*. (qPCRs n = 5, error bars are SEM). (**M**–**O**) 1 mg tamoxifen treated testes have visibly reduced immunohistochemical staining for the spermatid marker PGK 1/2 compared to controls whereas 250 µg tamoxifen-treated testes do not.

Our findings cannot exclude that the spermatogenic changes observed in the 3 mg tamoxifen treatment group are secondary to the delayed testicular descent induced by tamoxifen exposure as this is known to impact spermatogenesis and possibly endocrine function^37, 38^. To address this, we reduced the dose of tamoxifen used to a single dose of 1 mg or 250 µg (~12-fold less than that required to induce transgene expression), which pilot studies had shown did not induce delayed testicular descent. Consistent with this, the testes of mice treated with a single dose of 1 mg or 250 µg tamoxifen did not show a delay in testis descent compared to vehicle-treated controls (four out of four vehicle, five out of five 1 mg treated and five out of five 250 µg treated mice had descended testicles when examined at 5 weeks). However, as had been found with the 3 mg tamoxifen dose, mice treated on d16 with 1 mg or 250 µg groups both exhibited a significant reduction in SV weight and testis weight (although not body weight) at 7 weeks of age (five weeks after dosing) (Fig. 4a–c). No grossly visible reduction in spermatogenesis (Fig. 5A–C) or in seminiferous tubule diameter (Fig. 5D) was observed at 7 weeks of age in mice treated with 1 mg or 250 µg tamoxifen. Unlike the 3 mg treatment, there was no significant change in Sertoli cell volume per testis in either the 1 mg or 250 µg treatment groups (Fig. 5E), but the 1 mg tamoxifen-treated group had a significantly lower total germ cell volume per testis (Fig. 5F). Despite this overall change, the numbers of spermatogonia, spermatocytes, round and elongating spermatids per Sertoli cell were not significantly different between controls and tamoxifen-exposed animals (Fig. 5G–J). Despite this, testes from 1 mg tamoxifen treated animals showed significant reductions in the expression of both *Dkkl1* (Fig. 5K) and *Tnp1* (Fig. 5L) as well as visibly reduced immunohistochemical staining for PGK 1/2 compared to controls (Fig. 5M,O). Testes of mice treated with 250 µg tamoxifen showed a significant reduction in *Tnp1* (Fig. 5K), but not *Dkkl1* (Fig. 5L) but there was no conclusive change in the intensity of PGK 1/2 immunostaining (Fig. 5M–N). Together these data show that even extremely low exposure to tamoxifen continues to impact spermatogenesis several weeks later.

### A single dose of 1 mg tamoxifen alters Leydig cell maturation and function

**Figure 6:**
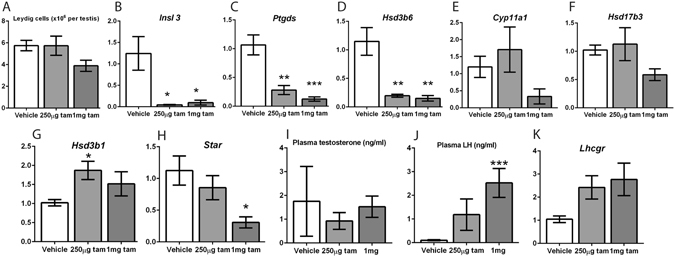
A single dose of 1 mg tamoxifen at d16 alters Leydig cell maturation and function in adulthood. (**A**) Leydig cell number per testis at 7 weeks was not significantly different to controls in both 1 mg and 250 µg INYF mice tamoxifen-treated mice (n ≥ 5, error bars are SEM). Expression of the maturation markers (**B**) *Insl3*, (**C**) *Ptgds* and (**D**) *Hsd3b6* is significantly decreased in both doses of treated mice compared to vehicle controls. Expression of the steroidogenic enzyme genes (**E**) *Cyp11a1* and (**F**) *Hsd17b3* are unchanged in tamoxifen-treated testes. (**G**) The expression of *Hsd3b1* is significantly increased in the 250 µg group but not the 1 mg group. (**H**) The expression of *Star* is significantly decreased in the 1 mg group but not the 250 µg group. (**I**) Plasma testosterone is not significantly different in treated mice compared to controls. (**J**) Plasma LH is significantly increased in the 1 mg (but not 250 µg) treated mice compared to controls. (**K**) The expression of *Lhcgr* in the testes of treated controls is also increased in the 1 mg but not 250 µg groups. (All qPCRs n ≥ 5, all hormone measurements n ≥ 7, error bars are SEM).

Since endocrine disruption in the testis may result in changes to Leydig cell maturation^39^, the number and maturation state of Leydig cells at 7 weeks after an injection of 1 mg or 250 µg tamoxifen at d16 was investigated. Although Leydig cell number per testis was not significantly altered (Fig. 6A), transcript expression of the established maturation markers *Insl3*, *Ptgds* and *Hsd3b6* ^39^ was significantly decreased in both groups of tamoxifen-treated mice compared to controls (Fig. 6B–D), demonstrating that even at extremely low single doses, tamoxifen is able to perturb testicular endocrine function many weeks later.

As Leydig cell maturation state can affect overall function and steroidogenic enzyme expression, the expression of steroidogenic enzyme genes was investigated at 7 weeks of age in 1 mg and 250 µg tamoxifen treated mice. Expression of *Cyp11a1* and *Hsd17b3* were not significantly altered (Fig. 6E–F), but expression of *Hsd3b1* was increased, though only in the 250 µg dose group (Fig. 6G), and the expression of *Star* was dose-dependently reduced (Fig. 6H). Plasma testosterone was not significantly different in either tamoxifen dose group compared to controls (Fig. 6I), but plasma LH was dose-dependently increased in the tamoxifen-exposed mice compared to controls (Fig. 6J), indicating compensated Leydig cell failure. The expression of *Lhcgr* in the testes of tamoxifen-exposed animals was also increased, although not significantly (Fig. 6K). Together these data demonstrate long term impacts on the endocrine function of the testes as a consequence of exposure to a single low dose of tamoxifen 5 weeks previously.

### A single dose of 1 mg tamoxifen changes the androgen/estrogen balance in the testis

**Figure 7:**
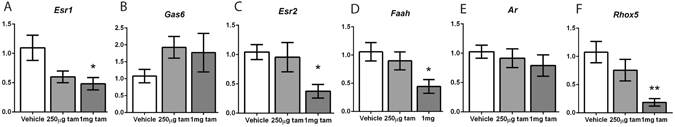
A single dose of 1 mg tamoxifen at d16 changes the androgen/estrogen balance in the testis. (**A**) Expression of the estrogen receptor α transcript *Esr1* is significantly decreased at 7 weeks after a single 1 mg (but not 250 µg) tamoxifen treatment at d16 in INYF mice. (**B**) *Gas6* is not significantly changed after either a 1 mg or 250 µg dose. (**C**) *Esr2* expression is significantly decreased after a 1 mg tamoxifen dose but not a 250 µg dose. (**D**) *Faah* expression is significantly decreased after a 1 mg tamoxifen dose but not a 250 µg dose. (**E**) *Ar* expression is not significantly different in either group. (**F**) *Rhox5* is significantly decreased in the 1 mg treated group but unchanged in the 250 µg treated group (All qPCRs n ≥ 5, error bars are SEM).

Both androgens and estrogens are important in normal testis function as is the balance between the two^40, 41^, and as tamoxifen is a SERM it could disrupt this balance. To address this, we investigated the expression of steroid nuclear receptor genes and their downstream target genes in the testes of tamoxifen-exposed mice, five weeks after exposure. Expression of *Esr1* was dose-dependently decreased by tamoxifen treatment (Fig. 7A). However, expression of the downstream Esr1 target gene *Gas6* was not significantly altered at either tamoxifen dose (Fig. 7B). Both *Esr2* expression (Fig. 7C) and expression of the downstream Esr2 target gene *Faah* (Fig. 7D) were significantly decreased in the 1 mg tamoxifen group compared to controls, but unchanged in the 250 µg dose group. Finally, *Ar* expression was not significantly altered in tamoxifen dose group (Fig. 7E), but expression of the downstream AR target gene *Rhox5* was significantly decreased in the 1 mg tamoxifen group (Fig. 7F). Taken together, these results demonstrate that a single low dose of tamoxifen in early pubertal life may significantly disrupt the androgen:estrogen balance within the testes in young adulthood.

## Discussion

There is an abundant literature that demonstrates that administration of estrogens to male rodents is able to induce both transient and long-term/permanent adverse changes to various reproductive organs and their functions, in particular impairment of spermatogenesis and steroid hormone production^42–44^. These effects are generally dose-dependent and can occur after exposure at various ages, although the nature and severity of effects may vary with the age of estrogen exposure, with perinatal exposure usually resulting in the most severe long-lasting consequences^42–44^. It seems that the most severe effects of estrogens in male rodents are mediated via ESR1 as deletion of this gene results in abrogation of the adverse estrogen effects, including on the testis^44^. Tamoxifen is a SERM with tissue-specific agonistic or antagonistic estrogen effects, but in the testis and generally throughout the male reproductive tract, tamoxifen acts as a potent estrogen agonist^30, 45, 46^. Consistent with this, tamoxifen administration is able to mimic at least some of the adverse male reproductive effects of estrogens described above^30, 45^, although these have not been studied in great detail and the effects in mice may vary between strains^45^. Tamoxifen is also thought to induce cellular stress in neurons, vasculature and ependyma by binding cholesterol epoxide hydrolase, highlighting that some detrimental effects of tamoxifen may not be mediated by estrogen receptors^47^. However this effect can be abrogated by delivery of tamoxifen in a vitamin E rich vehicle such as wheat germ oil.

The present findings, showing that early pubertal exposure of mice to a single dose of tamoxifen is able to dose-dependently induce adverse testicular effects, confirm and extend the earlier findings outlined above. However, the motivation for the present studies was not to add to this literature but to investigate the potential for tamoxifen induction of Cre recombinase in transgenic studies to induce adverse reproductive and endocrine functional changes in male mice that might be misinterpreted as resulting from transgene manipulation. Our findings show that such confounding effects of tamoxifen are likely to be a major issue for any study that involves evaluation of reproductive endpoints but also raises concerns where endpoint evaluation involves the study of tissues that are modulated by estrogens (or androgens) in view of the potential for both direct effects (of tamoxifen itself) and indirect effects (resulting from altered Leydig cell steroidogenic function in the testes); such effects may also vary between different strains^45^. We suggest below that appropriate controls are essential to guard against potential confounding effects.

The tamoxifen-inducible Cre system is a popular transgenic tool for controlling the disruption of gene expression at a specific time and in a specific cell type. However, despite a large number of studies suggesting that tamoxifen is an endocrine disruptor with long term effects not just on the male reproductive system but also on the wider body, our literature survey shows that many studies still do not include appropriate controls. In this study we show that a single early treatment of pubertal male mice with a dose of tamoxifen that is a third or even a twelfth of the mean single dose used for recombination induction in mice of this age group, can have long term effects on testis physiology and function. Moreover, each of the principle cell types of the testis (Sertoli, germ, Leydig) were adversely affected five weeks after dosing. Of particular concern is the persistent disruption of Leydig cell maturation and function after tamoxifen exposure, including possible resetting of the androgen/estrogen balance in the testis, based on the changes in steroid hormone receptor and target gene expression. Since most body tissues also express androgen and estrogen receptors and are exposed to the sex steroids produced by the testes, it is considered likely that a single dose of tamoxifen may also affect aspects of systemic physiology and endocrinology, some of which could be confounders in transgene induction studies.

Armed with this information, we suggest that transgenic studies using tamoxifen should be designed with six experimental groups: 1 [Cre positive tam-treated]; 2 [Cre negative (or floxed alone) tam-treated]; 3 [Cre postive floxed positive tam-treated]; 4 [Cre positive vehicle-treated]; 5 [Cre negative (or floxed alone) vehicle-treated]; 6 [Cre positive floxed positive vehicle-treated]. Whilst with hindsight and good research practices this study design appears obvious, as we have shown, the use of all six experimental groups is rarely seen in published studies, with 85 of the 206 primary research studies published in the past four years at risk of undocumented confounders arising from the impact of tamoxifen exposure. Worryingly, 15 of these 85 studies reported [Cre positive floxed positive vehicle-treated] as the only control, whilst 36 of the 85 did not report or show data from any controls whatsoever.

A further concern highlighted by this literature search is that, even in cases where a tamoxifen-treated control was employed, an equivalent vehicle-treated control was often absent. Without the additional use of vehicle controls alongside of tamoxifen only controls, correct interpretation of results obtained from gene deletion studies could be confounded, as the experimenter determines the impact of gene ablation in an entirely tamoxifen-exposed colony, rather than against normal physiological and endocrine conditions. The severities of off-target effects caused by tamoxifen are also likely to vary based on the mouse strain used^45^, the age of experimental animals, the estrogen (and androgen) responsiveness of the tissue of interest and the tamoxifen dose used. Therefore, it is essential to at least carry out a small pilot study with tamoxifen and vehicle controls to catalogue the off-target effects of tamoxifen and how they might impact on future studies.

Since the mice in our study were examined in early adulthood, we do not know whether the tamoxifen-induced changes that we report would persist and become worse as the mice age or whether they would resolve and the disrupted testis parameters return to normal. However, we consider the latter unlikely based on the extensive evidence of estrogen effects on the male^43, 44^ and, in general any adverse functions evident in young adult male rodents persist or worsen with age, rather than resolving^39^. In keeping with this, a study involving five consecutive daily injections of 3 mg tamoxifen from d50 to d54 in mice, found decreases in ventral prostate weight, Leydig cell *Insl3* expression and seminiferous tubule diameter, changes that were still evident 50 days later^48^.

In conclusion, this study identifies numerous caveats associated with the use of tamoxifen as an inducer of transgene expression, and highlights the acute sensitivity of the pubertal mouse testis to even a single low dose of tamoxifen. The adverse changes that are induced by such treatment are still evident many weeks after treatment, and by inference are likely to equally apply across other androgen and estrogen-sensitive tissues. These findings should be taken into consideration when planning experiments using this valuable transgenic tool.

## Materials and Methods

### Ethics Statement

All experimental procedures were approved by Edinburgh University Ethical Review Committee and conducted under licenced approval of the UK Home Office, project licence 60/4200, in compliance with the Animals (Scientific Procedures) Act, 1986.

### Mouse husbandry

Mice were kept in a 12-hour light/dark regimen with humidity maintained at 55% and temperature between 20 and 25 °C. Food and water were available *ad libitum*. Mice were fed a soy-free diet manufactured by Special Diet Services (SDS) and distributed by DBM (Scotland) Ltd.

### Transgenic lines used

Male congenic C57BL/6 J mice carrying a random insertion of either a non-inducible Nestin-Cre^49^, an inducible Nestin-Cre/ER^T2^ transgene^50^, or inducible iPDGFRa-Cre/ER^T2^ were used. Reporter lines used were R26R-YFP^51^ and R26R-Confetti^52^. Homozygous female R26R-EYFP mice were mated to male Nestin-Cre mice to produce the ‘NSYF’ line and male Nestin-Cre/ER^T2^ Cre recombinase mice to produce the ‘INYF’ line. Homozygous female R26R-Confetti mice were mated to male iPDGFRa-Cre/ER^T2^ mice to produce the IPRC line.

### Genotyping of animals

Mice were genotyped for inheritance of Cre Recombinase as previously described^53^. PCR amplification products were resolved using a QiaXcel capillary system (Qiagen, UK). An amplicon of 102 bp indicated inheritance of the Cre Recombinase transgene.

### Tamoxifen treatments

Tamoxifen (T5648, Sigma-Aldrich) solutions were made up to final concentrations of 1 mg/mL or 3 mg/mL. The powder was partially dissolved in sterile dimethyl sulfoxide (DMSO; D2650 Sigma-Aldrich) by gentle heating and vortexing, then diluted 1:10 in peanut oil (P2144; Sigma-Aldrich) and sonicated for full dissolution for a maximum of 5 minutes. Solutions were stored at −20 °C in opaque containers for a maximum of one month. Defrosted aliquots in use were stored concealed from light at 4 °C for a maximum of one week. Injections were administered intraperitoneally (i.p.) at 25 µl–200 µl depending on dose. Control animals were injected with 10% DMSO/90% peanut oil solution. Injections were performed blind to genotype.

### Recovery of tissues

Mice were euthanised by inhalation of carbon dioxide and subsequent cervical dislocation at 10:00 h. Testes were either fixed in Bouin’s fixative (Clin-Tech, Guildford, UK) for 6 hours or frozen on dry ice before being transferred to a −80 °C freezer for storage.

### Quantification of plasma hormone levels

Immediately after culling, blood was collected from mice by cardiac puncture with a syringe and needle pre-treated with heparin. Plasma was separated by centrifugation and stored at −80 °C. Testosterone was measured using an ELISA kit (DEV9911; Demeditec Diagnostics, Kiel, Germany). LH was measured using an in-house designed ELISA as previously described^54^. Samples for each treatment group or age group were analysed together.

### Histology and immunohistochemistry

Fixed tissues were processed and embedded in paraffin wax, and 5 μm sections were used for histological analysis. Sections of testis were stained with haematoxylin and eosin using standard protocols. Immunostaining was performed using a tyramide fluorescent immunostaining method as previously described^55^. Primary antibodies used were mouse α-nestin (Abcam ab6142), Rabbit α-GFP (Abcam #ab6556) or rabbit α-Pgk1/2 (Santa Cruz sc-28784).

### Stereological quantification of Leydig cell numbers

To determine the numbers of 3βHSD-positive Leydig cells per testis, the point-counting method was used as described previously^56, 57^. Image-Pro 7.0 with Stereologer plug-in software (MagWorldwide; Wokingham, UK) was used to visualise the immunostaining on-screen and carry out stereological quantification. A Leitz × 60 objective fitted to a Zeiss Axio-Imager microscope (CarlZeiss Ltd; Welwyn Garden City, UK) fitted with a HVC20 camera (Hitachi Denshi Europe, Leeds, UK) was the hardware used in this process. 3βHSD immunostaining was used to identify Leydig cells (LCs).

### Stereological quantification of germ cell numbers

Standard stereological techniques involving point counting of cell nuclei were used to determine the nuclear volume per testis of Sertoli cells and different maturational stages of germ cells, as previously described^58^.

### Stereological quantification of seminiferous tubule diameter

Seminiferous tubules were measured using a previously published method^53^. Only tubules with circular cross-sections were measured and measurements from 50 tubules per animal were recorded to have an accurate representation of seminiferous tubule diameter measurements.

### Preparation of testis cDNA

RNA was isolated from frozen testes using the RNeasy Mini extraction kit with RNase-free DNase on-the-column digestion kit (Qiagen, Crawley, UK) according to the manufacturer’s instructions. Due to changes in testis cell composition and size during normal development or due to the impact of gene deletion/treatment, 5 ng of luciferase mRNA (Promega, UK) was added per testis prior to RNA extraction as an external control to allow for direct comparison between samples^59^. RNA was quantified using a NanoDrop 1000 spectrophotometer (Thermo Fisher Scientific, Waltham, MA, USA) andcDNA was prepared using the SuperScript VILO cDNA synthesis kit (Life Technologies) according to manufacturer’s instructions.

### Quantitative RT-PCR (qPCR)

qPCR was performed on testis cDNA using an ABI Prism 7900 Sequence Detection System (Applied Biosystems) and the Roche Universal Probe library (Roche, Welwyn, UK). Roche UPL probes are labelled with 6-carboxyfluorescein (FAM) and were run as a multiplex with a luciferase assay^59^ in which the probe is labelled with NED. The assays for *Lhcgr, Cyp11a1, Hsd3b1, Hsd3b6, Hsd17b3, Cyp19a1, Insl3, Ptgds* ^39^ and *Rhox5* ^58^ have been previously published. New assays are detailed in Table 1. Resulting data were analysed using the ΔΔCt method^60^. All gene expression data was expressed relative to Luciferase mRNA expression. In the cases where Leydig cell-counts were carried out, relative mRNA expression was then corrected for Leydig cell numbers for Leydig cell-specific genes.

**Table 1:** Primer sequences and probes used for qPCR.

Gene	Forward primer	Reverse primer	UPL
*Ar*	ttatgaagcagggatgactctg	gctgccagcattggagtt	12
*Esr2*	cctcagaagaccctcactgg	cacgcacttcccctcatc	56
*Faah*	gcaggtgggctgttcagt	ccaagcagggatccacaa	83
*Star*	ttgggcatactcaacaacca	acttcgtccccgttctcc	11
*Dkkl1*	gaacacctccgggttcct	tccaggtctcgtagcaggtc	110
*Tnp1*	cggaagagcgtcctgaaa	agtccccctctgatgtcctc	51

### Statistical analyses

All analyses were performed using GraphPad Prism version 6 (GraphPad Software Inc, San Diego, CA, USA). Values are expressed as means ± SEM. Distribution of collected data was determined by a D’Agostino-Pearson normality test. If data was normally distributed, a two-tailed, unpaired t-test was used to compare two groups, unless a specific hypothesis was being tested, in which case a one-tailed test was used. Non-parametric data were compared using a Mann-Whitney U test. For 3 or more normally-distributed groups, a one-way analysis of variance (ANOVA) test followed by the Bonferroni post-hoc test was carried out. The Kruskal–Wallis test was carried out for 3 or more groups in case of non-parametric data. For analysis of cryptorchidism rates, Fisher’s exact test was performed.

## Electronic supplementary material

Supplementary table 1
