# Supplementary material for: Low-dose tamoxifen treatment in juvenile males has long-term adverse effects on the reproductive system: implications for inducible transgenics
Source: Sci Rep. 2017 Aug 21;7:8991. doi: 10.1038/s41598-017-09016-4 (PMC5566418; doi:10.1038/s41598-017-09016-4)
Supplement: Supplementary file 1 — Supplementary table 1 [file 41598_2017_9016_MOESM1_ESM.doc]

**Low-dose tamoxifen treatment in juvenile males has long-term adverse effects on the reproductive system: implications for inducible transgenics**

Saloni H. Patel, Laura O’Hara, Nina Atanassova, Sarah E. Smith, Michael K. Curley, Diane Rebourcet, Annalucia L. Darbey, Anne-Louise Gannon, Richard M. Sharpe, and Lee B. Smith

**Supplementary Data 1: Tamoxifen literature review**

Where multiple dose regimens have been used to attempt to induce a transgene, the concluded preferred dose of the investigators is reported.

Where a dose/weight is given in a reference but not an absolute dose, we assume weights given for C57bl/6J mice in Gall and Kyle, 1968 to calculate an absolute dose.

| **Embryonic (injection to mother, weight assumed to be 25g incl pups)** | | | | |
| --- | --- | --- | --- | --- |
| **Paper** | **Age** | **No. of**  **doses** | **Dose** | **Total dosage** |
| Zhang 2005 | Embryonic | Single | 1mg, 2mg and 3mg/40g | 0.625, 1.25 and 1.825mg |
| Danielian 1998 | Embryonic | Single | 1mg | 1mg |
| Furuyama 2011 | Embryonic | Single | 4mg | 4mg |
| Guo 2002 | Embryonic | Single | 4mg-10mg | 4mg-10mg |
| Hayashi 2002 | Embryonic | Single | 3mg | 3mg |
| Hodge 2013 | Embryos | Single | 180 mg/kg | 4.5mg |
| Laugwitz 2005 | Embryonic | 2 | 75ug/g | 3.75mg |
| Leone 2003 | Embryonic | Single | 1mg | 1mg |
| Nakamura 2006 | Embryonic (pre e13.5) | Single | 1.5mg | 1.5mg |
| Nakamura 2006 | Embryonic (post e13.5) | Single | 6mg | 6mg |
| Undeutsch 2014 | Embryonic | 5 | 1mg | 5mg |
| Sohal 2001 | Embryonic | 4 | 20mg/kg | 2mg |
| Qin 2008 | Embryonic | Single | 2mg | 2mg |
| Monvoisin 2006 | Embryonic | 3 | 1mg | 3mg |
|  | | | | |
| **Neonatal (injection to lactating mother)** | | | | |
| **Paper** | **Age** | **No. of doses** | **Dose** | **Total dosage** |
| Leone 2003 | Lactation to pups | 5 | 1mg | 5mg |
| Hirrlinger 2006 | Lactation to pups | 8 | 1mg | 8mg |
| Dhawan 2015 | Lactation to pups | 3 | 5mg (every 48h) | 15mg |
|  | | | | |
| **Neonatal (up to ~1 week)** | | | | |
| **Paper** | **Age** | **No. of**  **doses** | **Dose** | **Total dosage** |
| Chow 2006 | p0 | 1-3 | 3–4 mg/40 g | 87.5ug to 262.5ug (assume pup weighs 1g) |
| Furuyama 2011 | p1 | Single | 1mg | 1mg |
| Furuyama 2011 | p1 | Single | 1mg | 1mg |
| Sakamoto 2014 | p1 | Single | 600ug | 600ug |
| Claxton 2008 | OHT was administered via  intraperitoneal injection (20 lg per newborn pup) and  tamoxifen via gavage (3 mg per adult). In the tumor  model, a 1-mg tamoxifen pellet was implanted subcuta-  neously  OHT was administered via  intraperitoneal injection (20 lg per newborn pup) and  tamoxifen via gavage (3 mg per adult). In the tumor  model, a 1-mg tamoxifen pellet was implanted subcuta-  neously  p3 | Single | 20ug | 20ug |
| Acharya 2011 | p5 | 2 | 0.1 mg/g body weight | 600ug (assume pup weighs 3g) |
| Aloisio 2014 | p5 | 3 | 200ug | 600ug |
| Zheng 2014 | p5 | Single | 9 mg/40 g body weight | 675ug (assume pup weighs 3g) |
| Yoshida 2006 | p5 | 2 | 40 mg/kg | 240ug (assume pup weighs 3g) |
| Furuyama 2011 | p7 | Single | 1mg | 1mg |
| Furuyama 2011 | p7 | Single | 1mg | 1mg |
| Sultana 2014 | p8 | Single | 0.5mg | 500ug |
|  |  |  |  |  |
| **Juvenile (1-3 weeks)** |  |  |  |  |
| **Paper** | **Age** | **No. of**  **doses** | **Dose** | **Total dosage** |
| Chen 2007 | For analyzing the postnatal activity of the Cre recombi-  nation, 2-week-old mice received intraperitoneal injec-  tion of 1 mg of tamoxifen for 5 consecutive days  For analyzing the postnatal activity of the Cre recombi-  nation, 2-week-old mice received intraperitoneal injec-  tion of 1 mg of tamoxifen for 5 consecutive days  2 weeks | 5 | 1mg | 5mg |
| Nakamura 2006 | 2 weeks | Single | 2mg | 2mg |
| Furuyama 2011 | 2 weeks | Single | 2mg | 2mg |
| Qin 2008 | 2 weeks | 5 | 1mg/50g | 700ug (assume pup weighs 7g) |
| Zhu 2008 | 2 weeks | 5 | 1mg | 5mg |
| Sakamoto 2014 | 3 weeks | Single | 4mg (orally) | 4mg |
| Pazirandeh | 3 weeks + | 5 | 0.1mg/g | 5mg (assume mouse weighs 10g) |
|  | | | | |
| **Young adult (3-8 weeks)** | | | | |
| **Paper** | **Age** | **No. of**  **doses** | **Dose** | **Total dosage** |
| Weber 2002 | 4 weeks | 5 | 1mg | 5mg |
| El Marjou 2004 | 4 weeks | 5 | 1mg | 5mg |
| Lopez 2015 | 4 weeks | 5 | 75 mg/kg | 4.5mg (assume mouse weighs 12g) |
| Weber 2002 | 4 weeks | 5 | 1mg | 5mg |
| Sultana 2014 | 4-6 weeks | 5 | 0.25 mg/g | 15 to 20 mg (assume mouse weighs 12 to 16g) |
| Yang 2006 | 5-6 weeks | 10 | 0.5mg | 5mg |
| Zhang 2005 | 4-8 weeks | 5 | 2mg/40g | 3 to 5mg (assume mouse weighs 12 to 20g) |
| Seibler 2014 | 4-8 weeks | 5 | 5mg | 25mg |
| DeCarolis 2013 | 6-8 weeks | 5 | 180 mg/kg/day | 14.4 to 18mg (assume mouse weighs 16 to 20g) |
| Dor 2004 | 6-8 weeks | 5 | 8mg | 40mg |
|  | | | | |
| **Adult (>8 weeks, assumed weight 25g)** | | | | |
| **Paper** | **Age** | **No. of**  **doses** | **Dose** | **Total dosage** |
| Aloisio 2014 | Adult | 3 | 2mg | 6mg |
| Zhang 2013 | Adult | 7 | 2mg/20g | 17.5mg |
| Young 2008 | Adult | 5 | 0.25mg/g (oral) | 31.25mg |
| Weber 2003 | Adult | 5 | 1mg | 5mg |
| Willems 2011 | Adult | 5 | 1mg | 5mg |
| Willems 2011 | Adult | 5 | 3mg | 15mg |
| Wang 2009 | Adult | 4 | 9 mg per 40 g | 22.5mg |
| Undeutsch 2014 | Adult | 5 | 1mg | 5mg |
| Undeutsch 2014 | Adult | Single | 3mg | 3mg |
| Sakamoto 2011 | Adult | 4 | 10mg (oral) | 40mg |
| Claxton 2008 | Adult | Single | 3mg | 3mg |
| Dworniczak | Adult | 5 | 0.5mg/40 g | 1.56mg |
| Furuyama 2011 | Adult | Single | 4mg | 4mg |
| Furuyama 2011 | Adult | Single | 1mg | 1mg |
| Furuyama 2011 | Adult | 5 | 4mg | 20mg |
| Hayashi 2002 | Adult | Single | 9mg/40g | 5.625mg |
| Hayashi 2002 | Adult | 5 | 3 or 9 mg per 40g body weight | 9.375mg or 28.125mg |
| Hirrlinger 2006 | Adult | 8 | 1mg | 8mg |
| Hirrlinger 2006 | Adult | 10 (5 days) | 1mg | 10mg |
| Hoesl 2008 | Adult | 5 | 1mg | 5mg |
| Jiang 2014 | Adult | 6 | 150 mg/kg | 22.5mg |
| John 2008 | Adult | 3 | 2mg | 6mg |
| Latinga-van Leeuwen 2006 | Adult | 5 | 5mg | 25mg |
| Leone 2003 | Adult | 5 | 1mg | 5mg |
| Leone 2003 | Adult | 10 (5 days) | 1mg | 10mg |
| Leone 2003 | Adult | 10 | 1mg | 10mg |
| Ma 2003 | Adult | Single | 0.36mg/g | 9mg |
| Matson 2010 | Adult | 2 (1 day) | 2mg | 4mg |
| Monvoisin 2006 | Adult | 5 | 2mg | 10mg |
| Pan 2012 | Adult | 7 | 5mg (oral) | 35mg |
| Qin 2008 | Adult | 5 | 1mg/50g | 2.5mg |
| Sohal 2001 | Adult | 4-6 | 20 mg/kg per day | 2mg to 3mg |
| Guo 2002 | Adult | Single | 6mg-10mg | 6mg-10mg |
| Chen 2014 | Adult | 2 | 9 mg per 40 g | 11.25mg |
| Huh 2012 | Adult | 3 | 2mg/20g | 7.5mg |
| Gonneaud 2016 | Adult | 5 | 1mg | 5mg |

Pregnant mice were administered a mean total dose of 2.8 mg tamoxifen to induce transgene expression in their embryos. 10/14 regimens using pregnant mice used single doses. Neonatal mice up to one week old were either dosed directly or their lactating mother was dosed and tamoxifen ingested by nursing. Neonatal mice administered tamoxifen directly were given a mean total dose of 0.62 mg tamoxifen. 8/12 of these studies used single doses. Juvenile mice between one and three weeks old (pre-weaning) were dosed singly in 3/7 studies (mean of 2.6 mg) and 5 times in 4/7 studies (in 3/4 of these cases the total was 5 mg, one outlier was at 0.7mg). Mice classed as young adults from 3-8 weeks old (from weaning to breeding age) received a mean of 12.7 mg total tamoxifen with a range from 4 mg to 40 mg. All of these 10 studies used multiple doses. Adult mice from 8 weeks and older received a mean of 22.7mg tamoxifen (with a range from 1.5 to 40 mg) and the majority of these studies (30/37) were also multiple doses.

References

1. Gall, G.A. and W.H. Kyle, *Growth of the laboratory mouse.* Theor Appl Genet, 1968. **38**(7): p. 304-8.

2. Zhang, H., et al., *Efficient recombination in pancreatic islets by a tamoxifen-inducible Cre-recombinase.* Genesis, 2005. **42**(3): p. 210-7.

3. Danielian, P.S., et al., *Modification of gene activity in mouse embryos in utero by a tamoxifen-inducible form of Cre recombinase.* Curr Biol, 1998. **8**(24): p. 1323-6.

4. Furuyama, K., et al., *Continuous cell supply from a Sox9-expressing progenitor zone in adult liver, exocrine pancreas and intestine.* Nat Genet, 2011. **43**(1): p. 34-41.

5. Guo, C., W. Yang, and C.G. Lobe, *A Cre recombinase transgene with mosaic, widespread tamoxifen-inducible action.* Genesis, 2002. **32**(1): p. 8-18.

6. Hayashi, S. and A.P. McMahon, *Efficient recombination in diverse tissues by a tamoxifen-inducible form of Cre: a tool for temporally regulated gene activation/inactivation in the mouse.* Dev Biol, 2002. **244**(2): p. 305-18.

7. Hodge, R.D., et al., *Tbr2 expression in Cajal-Retzius cells and intermediate neuronal progenitors is required for morphogenesis of the dentate gyrus.* J Neurosci, 2013. **33**(9): p. 4165-80.

8. Laugwitz, K.L., et al., *Postnatal isl1+ cardioblasts enter fully differentiated cardiomyocyte lineages.* Nature, 2005. **433**(7026): p. 647-53.

9. Leone, D.P., et al., *Tamoxifen-inducible glia-specific Cre mice for somatic mutagenesis in oligodendrocytes and Schwann cells.* Mol Cell Neurosci, 2003. **22**(4): p. 430-40.

10. Nakamura, E., M.T. Nguyen, and S. Mackem, *Kinetics of tamoxifen-regulated Cre activity in mice using a cartilage-specific CreER(T) to assay temporal activity windows along the proximodistal limb skeleton.* Dev Dyn, 2006. **235**(9): p. 2603-12.

11. Undeutsch, H., et al., *A mouse model with tamoxifen-inducible thyrocyte-specific cre recombinase activity.* Genesis, 2014. **52**(4): p. 333-40.

12. Sohal, D.S., et al., *Temporally regulated and tissue-specific gene manipulations in the adult and embryonic heart using a tamoxifen-inducible Cre protein.* Circ Res, 2001. **89**(1): p. 20-5.

13. Qin, J., M.J. Tsai, and S.Y. Tsai, *Essential roles of COUP-TFII in Leydig cell differentiation and male fertility.* PLoS One, 2008. **3**(9): p. e3285.

14. Monvoisin, A., et al., *VE-cadherin-CreERT2 transgenic mouse: a model for inducible recombination in the endothelium.* Dev Dyn, 2006. **235**(12): p. 3413-22.

15. Hirrlinger, P.G., et al., *Temporal control of gene recombination in astrocytes by transgenic expression of the tamoxifen-inducible DNA recombinase variant CreERT2.* Glia, 2006. **54**(1): p. 11-20.

16. Dhawan, S., et al., *DNA methylation directs functional maturation of pancreatic beta cells.* J Clin Invest, 2015. **125**(7): p. 2851-60.

17. Chow, L.M., et al., *Inducible Cre recombinase activity in mouse cerebellar granule cell precursors and inner ear hair cells.* Dev Dyn, 2006. **235**(11): p. 2991-8.

18. Sakamoto, M., et al., *Continuous postnatal neurogenesis contributes to formation of the olfactory bulb neural circuits and flexible olfactory associative learning.* J Neurosci, 2014. **34**(17): p. 5788-99.

19. Claxton, S., et al., *Efficient, inducible Cre-recombinase activation in vascular endothelium.* Genesis, 2008. **46**(2): p. 74-80.

20. Acharya, A., et al., *Efficient inducible Cre-mediated recombination in Tcf21 cell lineages in the heart and kidney.* Genesis, 2011. **49**(11): p. 870-7.

21. Aloisio, G.M., et al., *PAX7 expression defines germline stem cells in the adult testis.* J Clin Invest, 2014. **124**(9): p. 3929-44.

22. Zheng, Q.S., et al., *Wt1 deficiency causes undifferentiated spermatogonia accumulation and meiotic progression disruption in neonatal mice.* Reproduction, 2014. **147**(1): p. 45-52.

23. Yoshida, S., et al., *The first round of mouse spermatogenesis is a distinctive program that lacks the self-renewing spermatogonia stage.* Development, 2006. **133**(8): p. 1495-505.

24. Sultana, T., et al., *Mice depleted of the coxsackievirus and adenovirus receptor display normal spermatogenesis and an intact blood-testis barrier.* Reproduction, 2014. **147**(6): p. 875-83.

25. Chen, M., et al., *Generation of a transgenic mouse model with chondrocyte-specific and tamoxifen-inducible expression of Cre recombinase.* Genesis, 2007. **45**(1): p. 44-50.

26. Zhu, M., et al., *Tamoxifen-inducible Cre-recombination in articular chondrocytes of adult Col2a1-CreER(T2) transgenic mice.* Osteoarthritis Cartilage, 2008. **16**(1): p. 129-30.

27. Pazirandeh, A., et al., *Multiple phenotypes in adult mice following inactivation of the Coxsackievirus and Adenovirus Receptor (Car) gene.* PLoS One, 2011. **6**(6): p. e20203.

28. Weber, P., et al., *Germ cell expression of the transcriptional co-repressor TIF1beta is required for the maintenance of spermatogenesis in the mouse.* Development, 2002. **129**(10): p. 2329-37.

29. el Marjou, F., et al., *Tissue-specific and inducible Cre-mediated recombination in the gut epithelium.* Genesis, 2004. **39**(3): p. 186-93.

30. Lopez, I.P., et al., *Differential organ phenotypes after postnatal Igf1r gene conditional deletion induced by tamoxifen in UBC-CreERT2; Igf1r fl/fl double transgenic mice.* Transgenic Res, 2015. **24**(2): p. 279-94.

31. Yang, B., et al., *Mouse model of inducible nephrogenic diabetes insipidus produced by floxed aquaporin-2 gene deletion.* Am J Physiol Renal Physiol, 2006. **291**(2): p. F465-72.

32. Seibler, J., et al., *Rapid generation of inducible mouse mutants.* Nucleic Acids Res, 2003. **31**(4): p. e12.

33. DeCarolis, N.A., et al., *In vivo contribution of nestin- and GLAST-lineage cells to adult hippocampal neurogenesis.* Hippocampus, 2013. **23**(8): p. 708-19.

34. Dor, Y., et al., *Adult pancreatic beta-cells are formed by self-duplication rather than stem-cell differentiation.* Nature, 2004. **429**(6987): p. 41-6.

35. Zhang, R.R., et al., *Tet1 regulates adult hippocampal neurogenesis and cognition.* Cell Stem Cell, 2013. **13**(2): p. 237-45.

36. Young, P., et al., *Single-neuron labeling with inducible Cre-mediated knockout in transgenic mice.* Nat Neurosci, 2008. **11**(6): p. 721-8.

37. Weber, P., et al., *Temporally controlled site-specific mutagenesis in the germ cell lineage of the mouse testis.* Biol Reprod, 2003. **68**(2): p. 553-9.

38. Willems, A., et al., *The development of an inducible androgen receptor knockout model in mouse to study the post-meiotic effects of androgens on germ cell development.* Spermatogenesis, 2011. **1**(4): p. 341-353.

39. Wang, X., et al., *A luminal epithelial stem cell that is a cell of origin for prostate cancer.* Nature, 2009. **461**(7263): p. 495-500.

40. Sakamoto, M., et al., *Continuous neurogenesis in the adult forebrain is required for innate olfactory responses.* Proc Natl Acad Sci U S A, 2011. **108**(20): p. 8479-84.

41. Dworniczak, B., et al., *Inducible Cre/loxP recombination in the mouse proximal tubule.* Nephron Exp Nephrol, 2007. **106**(1): p. e11-20.

42. Hoesl, E., et al., *Tamoxifen-inducible gene deletion in the cardiac conduction system.* J Mol Cell Cardiol, 2008. **45**(1): p. 62-9.

43. Jiang, Y. and J. Hsieh, *HDAC3 controls gap 2/mitosis progression in adult neural stem/progenitor cells by regulating CDK1 levels.* Proc Natl Acad Sci U S A, 2014. **111**(37): p. 13541-6.

44. John, G.B., et al., *Foxo3 is a PI3K-dependent molecular switch controlling the initiation of oocyte growth.* Dev Biol, 2008. **321**(1): p. 197-204.

45. Lantinga-van Leeuwen, I.S., et al., *Transgenic mice expressing tamoxifen-inducible Cre for somatic gene modification in renal epithelial cells.* Genesis, 2006. **44**(5): p. 225-32.

46. Ma, W., et al., *Hepatic vascular tumors, angiectasis in multiple organs, and impaired spermatogenesis in mice with conditional inactivation of the VHL gene.* Cancer Res, 2003. **63**(17): p. 5320-8.

47. Matson, C.K., et al., *The mammalian doublesex homolog DMRT1 is a transcriptional gatekeeper that controls the mitosis versus meiosis decision in male germ cells.* Dev Cell, 2010. **19**(4): p. 612-24.

48. Pan, Y.W., et al., *Inducible and conditional deletion of extracellular signal-regulated kinase 5 disrupts adult hippocampal neurogenesis.* J Biol Chem, 2012. **287**(28): p. 23306-17.

49. Chen, M., et al., *Wt1 is involved in leydig cell steroid hormone biosynthesis by regulating paracrine factor expression in mice.* Biol Reprod, 2014. **90**(4): p. 71.

50. Huh, W.J., et al., *Tamoxifen induces rapid, reversible atrophy, and metaplasia in mouse stomach.* Gastroenterology, 2012. **142**(1): p. 21-24 e7.

51. Gonneaud, A., et al., *Distinct Roles for Intestinal Epithelial Cell-Specific Hdac1 and Hdac2 in the Regulation of Murine Intestinal Homeostasis.* J Cell Physiol, 2016. **231**(2): p. 436-48.
